# Ethics of early detection of disease risk factors: A scoping review

**DOI:** 10.1186/s12910-024-01012-4

**Published:** 2024-03-05

**Authors:** Sammie N. G. Jansen, Bart A. Kamphorst, Bob C. Mulder, Irene van Kamp, Sandra Boekhold, Peter van den Hazel, Marcel F. Verweij

**Affiliations:** 1 Centre for Sustainability, Environment and Health, National Institute for Public Health and the Environment, RIVM, P.O. Box 1, Bilthoven, 3720 BA The Netherlands; 2 Department of Social Sciences, Wageningen University & Research, Hollandseweg 1, Wageningen, 6706 KN The Netherlands; 3 International Network on Children’s Health, Environment & Safety (INCHES), Ellecom, the Netherlands; 4 Ethics Institute, Utrecht University, Janskerkhof 13a, Utrecht, 3512 BL The Netherlands

**Keywords:** Early detection, Screening, Risk factors, Prevention, Ethics, Scoping review, Environmental health, Public health

## Abstract

**Background:**

Scientific and technological advancements in mapping and understanding the interrelated pathways through which biological and environmental exposures affect disease development create new possibilities for detecting disease risk factors. Early detection of such risk factors may help prevent disease onset or moderate the disease course, thereby decreasing associated disease burden, morbidity, and mortality. However, the ethical implications of screening for disease risk factors are unclear and the current literature provides a fragmented and case-by-case picture.

**Methods:**

To identify key ethical considerations arising from the early detection of disease risk factors, we performed a systematic scoping review. The Scopus, Embase, and Philosopher’s Index databases were searched for peer-reviewed, academic records, which were included if they were written in English or Dutch and concerned the ethics of (1) early detection of (2) disease risk factors for (3) disease caused by environmental factors or gene-environment interactions. All records were reviewed independently by at least two researchers.

**Results:**

After screening 2034 titles and abstracts, and 112 full papers, 55 articles were included in the thematic synthesis of the results. We identified eight common ethical themes: (1) Reliability and uncertainty in early detection, (2) autonomy, (3) privacy, (4) beneficence and non-maleficence, (5) downstream burdens on others, (6) responsibility, (7) justice, and (8) medicalization and conceptual disruption. We identified several gaps in the literature, including a relative scarcity of research on ethical considerations associated with environmental preventive health interventions, a dearth of practical suggestions on how to address expressed concerns about overestimating health capacities, and a lack of insights into preventing undue attribution of health responsibility to individuals.

**Conclusions:**

The ethical concerns arising with the early detection of risk factors are often interrelated and complex. Comprehensive ethical analyses are needed that are better embedded in normative frameworks and also assess and weigh the expected benefits of early risk factor detection. Such research is necessary for developing and implementing responsible and fair preventive health policies.

**Supplementary Information:**

The online version contains supplementary material available at 10.1186/s12910-024-01012-4.

## Background

Early detection of disease risk factors contributes to identifying pathways to prevent disease onset or moderate disease course and thereby decrease associated disease burden, morbidity, and mortality [1, 2]. Making disease predictions based on early risk factors has proven notoriously difficult since most diseases develop through a complex interplay between an individual’s susceptibility or predisposition to a certain disease or disorder and specific environmental exposures over time [3, 4]. New research is pushing the boundaries of understanding the pathways and mechanisms by which various factors interact by utilizing recent advances in computational and biomedical sciences that allow for measuring, modelling, and analyzing increasingly large clusters of environmental factors and linking these to disease outcomes. Initiatives to map the human exposome, i.e. “every exposure to which an individual is subjected from conception to death” [5], aim to uncover new (clusters of) risk factors and corresponding pathways to disease. An example is the Equal-Life project that studies the long-term effects of physical and psychosocial risk factors on children’s mental health and cognitive development [6]. One major practical aim of such initiatives is to enable and strengthen preventive strategies by improving the precision and accuracy of detecting early risk factors and identifying (groups of) people at risk of future disease.

The potential benefits of avoiding disease onset and corresponding disease burden may be significant. However, prevention of disease by early detection of risk factors also raises ethical concerns. For example, false positive results can lead to unnecessary medical treatment (e.g. biopsies), and, detection techniques can themselves involve risks, as is the case with, e.g., colonoscopies [7]. Moreover, the mere offering of medical preventive interventions can burden people with worries and uncertainties about their health [8]. And labeling environments such as neighborhoods as ‘high-risk’ can have stigmatizing effects that may, for example, affect school careers [9, 10]. Even for many preventive actions that have an obvious positive impact on public health, such as vaccination, only small benefit is expected for each participating individual as most of the participants would never develop the disease or severe complications in their lifetime [11].^1^

Early detection of disease risk factors likewise invokes ethical concerns. However, the current literature on this subject provides a fragmented and case-by-case picture, and no systematic efforts have been taken to capture the overarching ethical considerations of early detection of disease risk [12–15]. To improve this situation, the present paper presents a scoping review conducted with the dual aim of (1) providing an overview of the relevant ethical themes related to the early detection of disease risk factors, and (2) identifying potential gaps in the literature. The scoping review method allows for addressing a broad research question and including literature from different study domains and designs. In addition, the scoping review methodology allows for the broad mapping and thematically synthesizing of information, rather than solely summarizing the results [16], which makes it suitable for our aims.

This scoping review results in a summary of the characteristics of the included studies and an overview of common ethical themes as discussed in the literature, followed by a discussion of gaps in the literature. These results aim to guide future initiatives into detecting early risk factors and might thus be useful for ethicists, health practitioners and policymakers working in preventive medicine.

## Methods

A scoping review of the ethics literature was performed according to Arksey & O’Malley’s methodological framework [16], using the update by Levac et al. [17]. This review framework includes five main stages that are described below. Furthermore, the PRISMA-ScR guidelines established by Tricco et al. [18] and the PAGER reporting guidelines by Bradbury-Jones et al. were consulted [19].

### Identifying the research question

The aim of this study was to analyze the ethics literature on early detection of disease risk factors, and to define prominent ethical themes. Our assumption was that identifying such ethical themes could guide new developments in prevention such as exposome research and policies.

### Identifying relevant studies

Before conducting the systematic searches, Google Scholar was used to gather information for determining the appropriate scope, search terms, and feasibility of the search strategy. Two searches were conducted, in Scopus and Embase. Scopus was chosen for its wide range of literature in a wide range of domains. Embase was chosen for its comprehensive coverage of biomedical literature. These searches were performed on April 5, 2022. A third search in the Philosopher’s Index was performed for the same time period in December 2023. Philosopher’s Index was chosen for its disciplinary focus on philosophy and ethics literature. Keywords related to the domain of early detection (e.g. “Early detection” OR “Preclinical detection” OR “Predict*”) were combined with keywords relating to risk factors (e.g. “Risk factor” OR “Protective factor” OR “Determinant”) or the domain of exposome (e.g. “Exposom*” OR “Multi-expos*” OR “*omic”) and combined with the general domain of ethics (“ethic*”). For the full search strategy, see the supplementary materials.

### Study selection

Articles were included if they (1) discussed ethics of early detection of disease risks, (2) concerned human health, (3) were peer-reviewed and published in academic journals, (4) were written in English or Dutch. Articles were excluded if they (1) discussed a disease risk that is solely genetic (no environmental component), (2) primarily discussed detection of clinical symptoms or predicting treatment response (rather than discussing risk factors that could lead to the development of disease). Although our focus was on early detection of disease *risks*, papers discussing detection of presymptomatic disease were also included given that the distinction is not clearcut. First, two reviewers (SJ and IVK) screened all articles for meeting the inclusion and exclusion criteria based on titles and abstracts. All articles were independently (blindly) reviewed; conflicts were resolved by a third reviewer (BK). The remaining articles were screened based on the fulltexts and independently reviewed for meeting the criteria by at least two reviewers (SJ, BK, and BM). Conflicts were resolved by deliberation between the three reviewers. Finally, the reference lists of the included articles were consulted for additional literature.

### Charting the data

For all relevant articles, information was extracted by two authors independently from each other (SJ all articles, BK and BM both half of the articles). The following information relating to the type of article was extracted using a spreadsheet: the aim of the article, the method (empirical or non-empirical), the discussed type of risk factor and measurement method, target population, context (e.g. clinical practice, public health, occupational setting), the disease, and the action perspective of detecting the risk factor (e.g. treatment or other intervention available). For all relevant articles the ethical issues that were discussed substantially were extracted and categorized into either a class of issues related to the individual or familial sphere (e.g. patient informed consent and the duty to share relevant test results with family members), or a class of issues relating to a broader population or societal level (e.g. the issue of medicalization). Ethical issues that were mentioned but not further elaborated or analyzed were noted in a separate column.

### Collating, summarizing, and reporting the results

We present our results in a thematic narrative form [16], supported by an overview of the main themes and subthemes in Table 1, and the main themes per article in Table 2. For the descriptive analysis, information about the country of the first author, date of publication, the discussed health domain, the broader context of the early detection of risk factors, and the methods were captured. For the thematic analysis, a conventional content analysis approach was used [20]. The descriptive ethical issues extracted from the included articles were inductively coded by SJ. Recurrent coding patterns were identified and grouped into themes and subthemes. In an iterative process, the codes and developing (sub)themes were discussed by the three authors until conceptual stability was reached for the themes. Although the identified (sub)themes presumably have distinct importance and applications for different diseases and in specific situations, the aim was to categorize the most discussed issues and identify the broader ethical themes that arise out of the comparison of these issues. Finally, from the identified themes, literature gaps were identified.

## Results

### Descriptive analytics

**Fig. 1:**
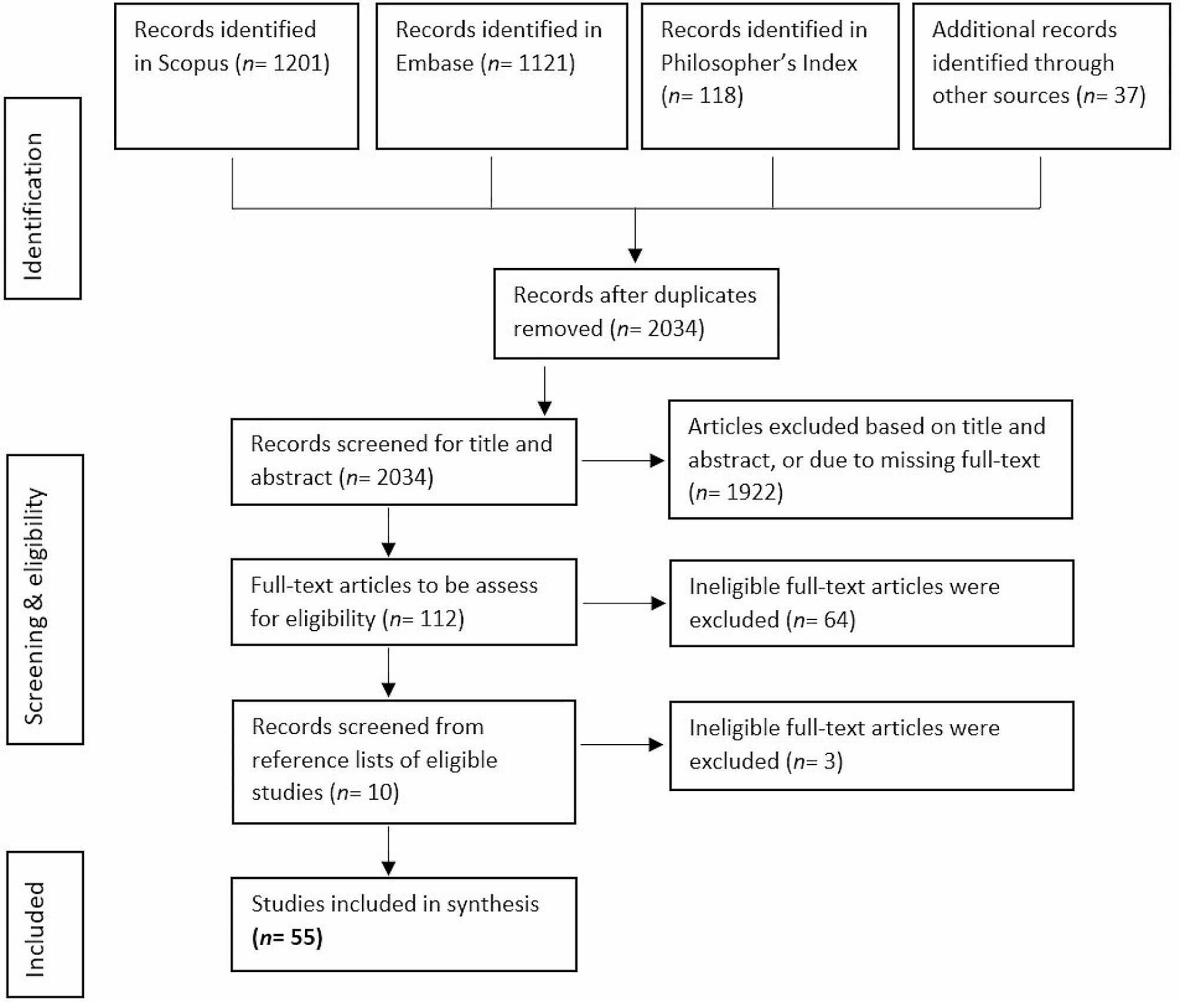
Preferred Reporting Items for Systematic Review and Meta-Analysis (PRISMA) flowchart of article screening phases

The searches resulted in *N* = 1201 articles from Scopus, *N* = 1121 articles from Embase, and *N* = 118 articles from Philosopher’s Index. Additionally, promising articles identified in the review’s preparational phase were added (*N* = 37). After removal of duplicates, *N* = 2034 articles were included for screening based on title and abstract. Abstract and title screening resulted in *N* = 112 articles that were found eligible for full-text reviewing. Full-text screening resulted in *N* = 48 articles fulfilling the criteria. Following the searches, we found further relevant articles (*N* = 7) by consulting the relevant articles’ references lists. A final sample of *N* = 55 was included in the analysis. See Fig. 1.

The included articles were published by authors in Europe (*N* = 31), the United States and Canada (*N* = 21), and Australia (*N* = 3). The articles were published between 1990 and 2021, with a peak between 2015 and 2019 (*N* = 18). A large part of the articles discussed the ethics of early detection of risk factors without focusing on a particular disease (*N* = 20) and many had a focus on mental health and neurological diseases (*N* = 25), followed by cancers (*N* = 8), nutrition (*N* = 2), and viral infection (*N* = 1). The articles discussed early detection of risk factors in the context of public health (*N* = 18), clinical health (*N* = 10), both public and clinical health (*N* = 15), occupational (*N* = 8) and forensic settings (*N* = 4). The majority of the included articles utilized methods common in applied ethics, including conceptual analysis and critical reflection on and engagement with the empirical literature, instead of conducting empirical research. The articles using empirical methods (*N* = 5) made use of focus group discussions [21, 22], interviews [23], ethnographic fieldwork [24], and expert workshops [14].

Analysis of the included articles identified eight common ethical themes: (1) Reliability and uncertainty in early detection, (2) Autonomy, (3) Privacy, (4) Beneficence and Non-maleficence, (5) Downstream burdens on others, (6) Responsibility, (7) Justice, (8) Medicalization and conceptual disruption. For an overview of these themes and the covered subthemes, see Table 1. The themes are in many ways interconnected, but for the sake of clarity will be discussed separately below. See Table 2 for the patterning chart of the main themes.

**Table 1:** Key ethical themes and subthemes for the early detection of disease risk factors

Main themes	Sub themes
Reliability and uncertainty in early detection	Validity, sensitivity, and specificity of the detection methods Predictive value and reliability of the detection methods Data biases The complexity of big data
Autonomy	Informed consent Health competencies Empowerment and responsibility for health
Privacy	Data protection Confidentiality
Beneficence and non-maleficence	Harmful psychological effects False-positive and false-negative results Actionability of test results Behavior and lifestyle change Risk communication
Downstream burdens on others	Changing perceptions of “at-risk individuals” Direct and indirect implications for family and significant others
Responsibility	Individual responsibilities for health Collective and societal responsibilities for health
Justice	Stigmatization and discrimination Health inequities Equitable and efficient use of financial resources
Medicalization and conceptual disruption	Reconceptualization of health and disease Overdiagnosis and overtreatment

**Table 2:** Key ethical themes and patterning chart

	Themes in the Ethics of Early Detection of Disease Risk Factors							
Paper ID	Reliability & uncertainty	Autonomy	Privacy	Beneficence & non-maleficence	Downstream harms to others	Responsibility	Justice	Medicalization & conceptual disruption
**Ahlgrim et al. 2019**	x	x		x		x	x	x
**Almond 2006**	x	x	x	x	x		x	x
**Bolt et al. 2021**			x	x		x	x	x
**Bunnik & Bolt 2021**	x	x	x	x		x	x	
**CalzÃ et al., 2015**	x			x			x	x
**Chowdhury et al. 2013**	x	x	x	x			x	
**Christiani et al. 2001**			x	x		x	x	x
**Corcoran et al. 2005**	x	x	x	x	x		x	
**DeCamp & Sugarman 2004**	x		x	x		x	x	
**Dhondt 2010**	x	x		x		x	x	x
**Frank 1996**				x			x	
**Frank 2001**		x	x	x		x	x	
**Gershon & Alliey-Rodriguez 2013**		x	x	x	x	x	x	
**Glenn 2019**	x		x	x	x		x	
**Green & Hillersdal 2021**		x		x		x	x	x
**Green & Vogt 2016**	x	x	x	x		x	x	x
**Hall et al. 2014**	x	x	x	x	x		x	x
**Hall et al. 2004**				x	x	x	x	
**Hall et al. 2008**			x	x		x	x	x
**Hoge & Appelbaum 2012**	x	x	x	x	x	x	x	
**Holzman 1996**	x	x	x				x	
**Horstkötter et al., 2021**		x				x	x	x
**Hurlimann et al. 2017**		x	x	x		x		x
**Illes et al. 2007**		x	x	x	x	x	x	x
**Jenkins et al. 2008**	x	x	x		x			
**Jurjako et al. 2019**	x	x		x		x	x	x
**Lawrie et al. 2019**	x	x	x	x			x	
**Lewis 2018**		x	x			x	x	
**Meager et al. 2017**		x	x	x		x	x	x
**McKeown et al. 2021**	x	x		x		x	x	x
**Paul et al. 2014**	x	x		x		x	x	x
**Plutynski 2012**	x	x	x	x			x	x
**Prainsack 2019**			x	x		x	x	x
**Press et al. 2000**		x		x		x		
**Quattrocchi et al. 2019**		x		x			x	
**Rawbone 1999**	x	x	x	x			x	
**Roberts et al. 2013**		x		x			x	
**Salamanca-Buentello et al. 2020**	x			x		x	x	
**Schermer & Richard 2019**				x	x		x	x
**Schicktanz et al. 2014**		x	x	x	x	x	x	x
**Singh & Rose 2009**	x	x	x	x	x		x	
**Specker & Schermer 2021**	x		x	x			x	
**Specker & Schermer 2017**	x			x	x	x	x	
**Spriggs et al. 2008**	x	x		x	x	x	x	
**Stol et al. 2016**		x	x	x		x	x	x
**Stol et al. 2017**	x	x		x		x	x	x
**Stol et al. 2018**	x	x	x	x		x	x	
**Svensson & Sandlund 1990**		x		x		x	x	
**Tabery 2009**	x	x		x			x	
**Thomas 2015**	x	x	x				x	
**Tromp et al., 2021**	x							x
**Van Damme et al. 1995**	x	x	x	x		x	x	
**Vineis 1997**		x	x	x		x	x	
**Vineis & Schulte 1995**	x	x	x	x			x	
**Vogt et al. 2019**	x	x		x				x

### Reliability and uncertainty in early detection

Reliability and uncertainty of early risk information are frequently discussed as important ethical considerations for detecting early disease risk factors [see Table 2, column Reliability and uncertainty]. The efficacy and accuracy of detecting the risk that is investigated are, for example, often discussed. Where diagnostic tests provide binary outcomes (a disease is present or not present), the factors detected with methods to determine and predict disease risk provide probabilistic outcomes from 0 to 100% risk for the disease to develop, where the low and high extremes are very rare for most diseases as many biological and environmental factors affect the risk score [4, 15, 25].

In risk screening tests, reliability and uncertainty are often discussed in terms of validity, i.e. sensitivity and specificity, of the test [26, 27]. Sensitivity refers to the chance that the test returns a positive result in people that are at risk (true positive rate) whereas specificity refers to a negative result in people that are not at risk (true negative rate). Tests with a low validity, therefore, return more false-positive and false-negative results. The predictive value of a test also depends on the disease prevalence in the target population. For example, if the prevalence of a risk factor is very low, even a test with high sensitivity and specificity will have a low positive predictive value. Furthermore, the analytical validity of the test does not necessarily mean clinical validity and utility, i.e. how well the test correlates with clinical responses and treatments [21]. Depending on these latter two, the number of at-risk people that need to be correctly detected and treated to prevent one person from developing the disease (“number needed to treat”) varies for different diseases and detection methods [28].

As preventive medicine is turning towards detecting and preventing more complex, multifactorial diseases, authors^2^ warn that the predictive value and reliability of the detection methods need to be carefully monitored and balanced against other aspects such as the cost-effectiveness and actionability of the risk information (see the sections on, respectively, justice and beneficence & non-maleficence). Furthermore, non-genetic risk factors, including epigenetic factors [21, 29, 30], can change over time and interactions with different environmental factors and conditions can produce different outcomes. This increases the uncertainty associated with risk predictions [4, 25, 27, 31, 32], but also provides opportunities for preventive interventions to decrease risk dispositions and modify the disease course [43].

Early detection of disease risk factors can be affected by, and play into, various biases that decrease the reliability of test results and increase uncertainty in risk predictions. Frequently mentioned is the way in which selection bias in the development and validation phases of detection methods (e.g., due to non-representative study samples) can limit the generalizability of these detection methods [33, 34]. In the other direction, the ability to detect risk factors and abnormalities in increasingly early phases can play into lead-time bias (where earlier detection leads to a mistaken sense of increased survival time), length bias (where the effectiveness of a test is overestimated because it over-identifies slow-developing, less aggressive diseases), and overdiagnosis bias (where many people are identified as high risk for a disease that will never develop during their lifetime [35]). (For overdiagnosis, also see the section on medicalization and conceptual disruption.)

To overcome potential biases and improve the predictive value and reliability of early screenings the use of big data approaches is sometimes proposed as a solution. However, authors warn that “it is not always the case that more data will lead to better predictive models” [28, p. 124] as it can also increase the complexity and degree of uncertainty by increasing the variance of the results (and thereby causing loss of precision). Moreover, it does not necessarily solve other issues discussed in this section or the thematic sections below [24, 36, 37].

### Autonomy

A commonly mentioned set of issues centers around the notion of personal autonomy [see Table 2, column Autonomy], which we understand here in the broad sense of being able to “lead one’s life in a way that accords with what one genuinely cares about” [38, p. 5]. In the surveyed papers, autonomy considerations about having the capacities and opportunities to make one’s own choices are mostly discussed in the context of informed consent. There is broad consensus among authors that informed consent should contain information about the expected benefits and possible medical and psychosocial risks [39], about who can use and access the data (e.g. secondary uses by third parties [40]), and about the possibility of incidental findings that may be sensitive as they might unveil environmental and lifestyle exposures [21]. The extensive and complex nature of the information required for truly informed consent, however, requires a level of health literacy that for many individuals may not be attainable [23]. For example, worries are expressed that complex health information can overwhelm people and compromise their capacity for autonomous decision making [41], that it is difficult for many people to understand the difference between absolute and relative risk [35], and that people might have unrealistic ideas about the explanatory power of early disease risk factors [42].

Concerns about (lack of) health-related competencies are especially prominent in the surveyed literature. Dilemmas can arise when early detection takes place early in life and consent had to be given by legal proxies (parents or legal guardians). One issue here is that preventive testing in children might deprive them of their ‘right not to know’, in which case it could be preferable to postpone testing until the young person has developed sufficient competency to make their own decision. However, waiting can also deprive the same person of the opportunity to make choices that can affect their disease risk, or compromise their health and potentially the development of necessary competencies by allowing the disease to develop [13, 15, 30, 39, 43–45]. Competencies for informed consent in adults is mostly discussed for individuals atrisk of mental health disorders [39, 46]. Developing mental disease symptoms can increasingly compromise the required competencies such that “a fully competent and autonomous patient at the beginning of a study may progress to a point of diminished capacity and autonomy” [12, p. 7].

Another subtheme related to autonomy that several authors critically discuss is empowerment, in particular the idea that early detection can empower people to take control of their health and to plan their future. It is also discussed that there is a risk that the underlying assumption is that individuals “can (and should) be held morally responsible for their health outcomes” [47, p. 77]. As social and moral norms promoting responsibility for health can put pressure on individuals and groups to conform, several authors worry that the narrative of empowerment might compromise the voluntariness of the decision to take an early detection test [28, 29, 48–51], as well as downstream decisions about lifestyle choices [32].

### Privacy

Early detection of health-related risks generates sensitive information about a person’s susceptibility to a variety of diseases [see Table 2, column Privacy]. Moreover, the information that is collected in the service of such an assessment can potentially contain indicators of a person’s (past) lifestyle and environmental exposures that also warrant protection (e.g. via epigenetic changes) [4, 21, 29]. Therefore, *confidentiality* of tests and results is considered an important component of protecting sensitive data and preserving individual privacy, but ensuring confidentiality becomes increasingly difficult when a broad range of data is collected and possibly shared or linked to other (public) data sources [4, 21]. Linking datasets increases the risk of identification of individuals in the datasets [4, 14, 21, 22]. Pooling or aggregating data before sharing reduces the risk of identification, but also decreases the richness of the dataset and its (clinical) utility [15].

For the informed consent procedure (also see the section Autonomy), clarity about individual privacy, data confidentiality, and data storing and sharing are frequently mentioned [13, 21, 33, 42, 44, 52, 53]. As individuals might worry about (future) disclosure of their risk information, information about potential risks to privacy and how institutions deal with potential privacy breaches are important for valid informed consent. A lack of confidentiality, or a lack of trust in confidentiality, might lead to people not participating in early detection efforts that can benefit them [54, 55]. Legislation to protect sensitive personal information can provide reassurance [33].

Some authors hold that confidentiality might be rightfully breached in certain cases, such as when parents are acting as a proxy for their child [15] or when the risk information is relevant to others as well, such as (future) caretakers and family members who potentially carry the same risk factors. However, hesitance was observed in the literature with respect to assigning a moral duty to physicians or at-risk individuals to share a risk status with relevant others as this would harm their right to autonomy [44, 45, 52].

Broad consensus was observed about the importance of protecting privacy and confidentiality against third parties such as insurers and employers. Worries exist that third parties can misuse risk information to discriminate or stigmatize individuals who have been labeled as being high risk of disease [13, 27, 30, 35, 43, 48, 56, 57]. (See also the section on Justice.)

### Beneficence and non-maleficence

In healthcare, the principles of *beneficence* (“do as much good as possible”) and *non-maleficence* (“do no harm beyond what is proportionate”) are important moral guiding principles [58] for striking a positive balance between an intervention’s benefits and inflicted harms for the individual. While the principles themselves are mentioned relatively infrequently in the surveyed literature (but see [27, 35, 40]), they are implicitly present in the background of many discussions about the potential benefits and harms of the early detection of disease risks [see Table 2, column Beneficence & non-maleficence]. For example, multiple authors argue that the ‘latent period’ between detecting a risk and potential disease occurrence can also be a period of uncertainty and anxiety. They question whether early knowledge about being at heightened risk is more beneficial to an individual than spending the interim time in ‘normalcy’, especially when no preventive actions are currently available [13, 32, 39].

Most discussed are the ways in which a high-risk classification may lead to worries and anxieties for developing disease [2, 13, 22, 23, 28, 32, 44–48, 59] and can have negative effects on self-image [22, 31, 32, 45, 46]. Authors note that the effects on self-image might lead to depression [33] and even suicide [12, 46], although these effects are generally considered rare [49]. Another possible harmful psychological effect might be that positive test results lead to a perceived lack of control and decreased motivation for a future that “threatens to be taken away by illness”, possibly influencing important life decisions such as family planning [39, p. 6]. Knowing one is at greater risk for disease can also cause feelings of being fragile or ‘defected’ [4, 39, 54]. Such knowledge can also contribute to a self-fulfilling prophecy [12, 15, 31, 60] when the (anticipated) risk status leads to stress and anxieties that subsequently affects cognitive functioning [12], which in turn promotes risk-increasing behaviors [15].

Harmful impacts of early detection of disease risk factors are especially problematic and unjustified when results are incorrect. Authors warn that false-positive results can lead to unnecessary labeling and interventions [15, 27, 28, 39, 41, 44, 60]. Likewise, false-negative results can deprive patients from beneficial early interventions and provoke unjustified feelings of security [27, 28, 47, 60], possibly leading to the neglect of early symptoms (“They said everything was okey”) [2, p. 278]. (Also see the section Reliability and uncertainty). Advances in research methods, however, promise to increase the precision of screening tests and thereby decrease mis-categorizations [61].

In short, it is not always beneficial for individuals to participate in early detection programs and undergo (sometimes unnecessary) follow-up examinations and interventions [35]. Though transparency and truth telling by disclosing test results and possible incidental findings are valued as providing respect for autonomy, consensus within the medical community is at present against disclosure of risk information with uncertain predictive value, justified by the principle of non-maleficence [39, 49].

Many authors acknowledge that if early knowledge is to be beneficial to individuals, the screening results should be *‘actionable,’* in the sense that they present (viable) options open to individuals to change their situation and health prospects. The existence of an “effective intervention to prevent the disorder in those who are identified as being at risk” is even identified by some as a prerequisite for the ethical acceptability of early screening tests [57, p. 352], especially when it concerns children who cannot yet decide for themselves [45]. Apart from early interventions to fully prevent disease occurrence several other preventive actions are mentioned that can be considered important for beneficence, including providing reassurance when a test is negative [14, 15, 46, 49], offering support in planning one’s life for a future disease [45, 46], supporting reproductive decisions [32, 41, 43, 62, 63], and giving advice on modifying health behaviors and lifestyle [15, 32, 41, 47, 51, 64].

Promoting healthy lifestyles and health-positive behaviors are mentioned in particular as important interventions to mitigate disease risk and support beneficence [12, 29, 32, 51, 59]. The assumption that individuals can and will successfully implement the provided lifestyle and health behavior advice is, however, questioned and criticized by several authors. Social science studies indicate that changing health behaviors is difficult [28] and the lack of direct experience of symptoms, uncertainty about whether symptoms will materialize, and uncertainty about the effectiveness of changing lifestyle are mentioned as possible demotivating factors [2]. The harmful psychological effects discussed above can also be barriers to effective behavior change [2, 4]. Even when risk information effectively motivates some individuals to change health behaviors, it should not be assumed to motivate a particular individual [43]. Other barriers to adopting healthy lifestyles that are mentioned are low health literacy, low socio-economic status, and restricted access to healthcare. The implications of these inequalities between individuals and social groups are discussed in the theme on Justice.

To achieve the proposed benefits and minimize the harms, adequate communication about risks is discussed as crucial. Participants of preventive interventions should be informed about, among other things, the expected benefits and harms and the actionability of the risk information (also see the section on informed consent within the theme Autonomy). It may be difficult, however, for both professionals and laypersons to adequately grasp the difference between susceptibility and disease, and to understand probabilistic and relative risk data [35, 39, 43, 50, 59]. Some authors recommend avoiding complex medical terminology and contextualizing the provided information in relation to the patient’s situation [12, 53]. Misinterpretation of test results and unsubstantiated expectations for the explanatory power and actionability of the information (*therapeutic misconception*) are widely discussed as harmful implications of inadequate communication of risk information [12, 42, 46, 47, 49, 59, 63]. An example by Schermer & Richard [53, p. 143] is that “the emotional and social effects of terms chosen to communicate with lay-people can be considerable; being told one is ‘at risk’ for developing AD [Alzheimer’s disease] is different from being told one has preclinical or asymptomatic AD – although the situations these terms aim to describe may be exactly the same”. Educating patients using simple support aids [45], offering counseling [39, 43], and training healthcare professionals in patient communication are discussed as benefitting risk communication [44, 49].

### Downstream burdens on others

Besides the harms and benefits of early risk factor detection for the individuals who consent to screening procedures, authors frequently mention the downstream effects that screening participation may have for, and in relation to, friends and family [see Table 2, column Downstream burdens on others]. Though these downstream effects to a certain extent relate to the bioethical principles of beneficence and non-maleficence as well (e.g., consider the social harms that may befall individuals through stigmatization; see also Justice), we discuss them separately because they also relate to broader ethical questions about how to strike a balance between diverging interests of multiple individuals.

In this context, authors mention that family and others around “at-risk individuals” might think of them “as in some sense already impaired” [43, p. 69] and treat them differently [42, 61, 63]. For example, children might be treated differently at school [39]. This does not need to be harmful per se, but authors warn that it can have adverse impacts on relationships [15], cause conflicts with the family [13, 45, 52], and contribute to possible self-fulfilling prophecies [15, 43, 53] (see also section Beneficence and Non-Maleficence).

To prevent conflicts or misunderstandings within families and relationships, providing adequate risk information and an explanation of what a disease risk means for the screened individual and relevant others is important. Multiple authors propose that family counseling should be offered when conflict is probable [39, 47, 52]. Risk information can also have direct consequences for relatives when the risk is inheritable or when parents need to take decisions on behalf of their child, for example. Interests of family or significant others can create tensions between the individual’s right to keep risk information confidential and opportunities to reduce risk for others (the principle of non-maleficence). This raises questions about the duties of the patient and his or her physician towards other persons at risk [40, 45, 52].

### Responsibility

Responsibilities for health outcomes and the development and prevention of illness are discussed in the majority of the included articles [see Table 2, column Responsibility]. As was touched upon in the themes of Autonomy and Beneficence and Non-maleficence, empowerment of people to use risk information to make health decisions and manage their well-being is an important driver for the early detection of disease risk factors. Although some authors mention the possibility that detection of risk factors, especially biological factors, might lead to ascribing decreased responsibility to individuals for ill health [4, 31], most authors discuss that individual responsibilities for health and well-being are increasing due to a focus on personalized disease risks, leading to individuals also increasingly being held accountable for their illhealth [22, 24, 28, 29, 32, 47, 50, 51, 55, 59, 62]. However, emphasizing individual responsibility for health and well-being might overburden individuals and suggest they are to blame for outcomes that are not always within their control. Such lack of control can be caused by the amount and complexity of health-related information [41] or the lack of means and resources to be proactive about health [37, 42, p. 204].

Authors warn that if such responsibility shifts towards the individual occur, individuals or parents who are not acting on health information or choose not to participate in early screenings might then elicit victim blaming [2, 21, 29, 34, 56, 65], and the people that do not comply with the norm of taking individual responsibility for health prevention might become seen and treated as “irresponsible” [22, 28, 29, 37]. Norms of individual responsibility for health can also lead to feelings of guilt and self-blame when disease occurs [21, 22, 62]. According to Singh & Rose: “if biomarkers for [antisocial behavior] are found to be present during early childhood screening, then children might be subject to intrusive medical interventions that focus on individual-level risk factors rather than on social and environmental risk factors.” [42, p. 204].

An increased focus on individual risk factors can indeed obscure environmental and societal factors for illhealth that are often more structural and affect larger populations. If these factors remain unnoticed this will push away responsibility of e.g. employers, industry, and governments for these risks [21, 29, 54, 56, 60]. Worries are expressed that this also shifts the focus away from studying and analyzing collective interventions to promote health [2, 62], whereas preventing disease by intervening in harmful environmental or societal factors, e.g. environmental pollution or norms of sedentary working, is often more effective and avoids blaming individuals [23, 29, 37, 57, 59]. In the occupational context, worries are expressed that this shift towards individual responsibility for health can lead to the blaming or discriminating of ill or at-risk employees instead of taking responsibility for safe working environments [26, 48, 56, 66].

### Justice

Many of the discussed considerations have implications for justice, which is here understood as the fair, equitable, and appropriate treatment of persons and fair distribution of healthcare resources [67] [see Table 2, column Justice]. As the prevalence of disease risks varies between individuals and groups of individuals, identifying individuals and groups at high risk can be used to support and target those who need help the most. However, there are concerns that the labeling of individuals or groups can also have stigmatizing and discriminatory effects [13, 14, 34, 40, 43, 51, 63, 68]. This is especially problematic in the absence of effective preventive interventions [32, 60], although it is also argued that, in the long run, preventive interventions “may reduce the severity of both security-based and shame-based stigma” [63, p. 217]. Special attention is paid to ethnic and societal groups that are currently, or historically have been, vulnerable to stigmatization and discrimination [21, 30, 39–42, 45, 54, 65, 69].

Ascribing responsibility and blame for ill health are described as a base for possible stigmatization and discrimination. This can take many forms, including social exclusion, reduced or denied access to healthcare services and insurance, and exclusion from educational institutions or jobs. Besides the detrimental effects of stigmatization and discrimination itself, fear of these effects might also generate self-stigma – causing harmful psychological effects and possible social withdrawal while the actual disease might never develop – and it might withhold people from participating in testing or screening programs that might benefit them [4, 54, 57, 68].

The distributive justice concerns that are discussed relate predominantly to inequalities in access to healthcare and opportunities to prevent disease. When early risk factors screenings are introduced, fair access to the testing and subsequent interventions should be endorsed as well as the provision of appropriate information for different societal groups. When this is not the case, health inequalities can exacerbate along socioeconomic gradients as not everyone can afford testing and follow-up healthcare, and, as within the theme of Responsibility, not everyone has the same capacities to understand risk information and take appropriate measures to protect their health [2, 14, 22, 29, 32, 37, 47, 51, 65]. The focus on individual responsibilities for health promotion and disease prevention are also argued to undermine health solidarity [22, 44].

Another concern is the fact that many biological and environmental risk factors are much more common in certain minority or otherwise disadvantaged groups than in less deprived populations [40, 65, 69]. These inequalities add to the above-mentioned inequality in access to healthcare and preventive opportunities that affect the same vulnerable and disadvantaged groups [37, 65]. Detecting risk factors in these vulnerable groups and individuals is argued to improve the understanding of what types of interventions work for different groups, thereby possibly contributing to disease prevention to achieve more health equity overall [61]. However, these risks should not be individualized in a way that harmful environmental and societal risk factors are neglected, as this could suggest that ultimately persons are themselves responsible for their disease – which could amount to victimblaming. Moreover, it could lead to diminished efforts by government and private organizations to tackle the structural social and environmental determinants underlying health inequities, possibly leading to decreased health solidarity and expanding health inequality [22, 65].

Mental health and neuropsychiatric disorders are discussed as current causes for stigma. Several authors have concerns that early detection of risks for mental health disorders will subject high-risk individuals to similar stigmatization, potentially extending to family members as well [15, 45, 52, 63]. Not only might those at high risk for mental health disorders experience stigma from the people around them, e.g. friends and family, or teachers, employers, or healthcare professionals, but they are also vulnerable to self-stigmatization and other harmful psychological effects, further increasing the risk of developing mental health pathologies [12, 39, 70]. (Also see the section Downstream harms on others.)

If tests for risk factors or knowledge about disease risk are used in occupational and insurance contexts this raises concerns about stigmatization and discrimination. Employers might use information on employees’ risk status to provide a safe work environment and protect their employees’ health. However, it can also result in a situation where employees (possibly unintentionally) favor workers who are less likely to develop illness. Testing for early disease risks in the workplace should be used to include people in the workplace by improving safe and healthy workplaces and not as a means for “selection of the fittest” [26, p. 98] or excluding people from the workplace [4, 21, 40, 43, 48, 49, 54–56, 59, 66, 69]. Insurers might use risk information for differentiating insurance premiums or excluding people from insurance. They might also impose pressure on people to accept tests, share their test results, or require clients to modify their lifestyles and environment based on their personal disease risks [4, 14, 44]. Regulations about privacy and confidential use of disease risk information are very important to protect people against stigmatization and discrimination. (See also the theme Privacy.)

Finally, there are concerns about the impact of early detection of disease risk on equitable and efficient use of financial resources for healthcare. Questions are raised whether screening interventions produce sufficient health benefits given the healthcare budget they require [13, 37, 41, 44, 49, 55]. Moreover, increasingly sensitive technologies will expand opportunities and needs for follow-up examinations, the monitoring of detected risks, and providing preventive treatments, therapeutic interventions, or counseling [28]. When taking into account these downstream effects, it is not obvious that early risk factor detection has a favorable cost-effectiveness ratio compared to clinical healthcare [37, 68]. Finally, commercial screening tests risk draining collective healthcare resources: individuals or companies may purchase such tests for themselves but follow-up examination and preventive treatments will subsequently be sought in the public healthcare system [23].

### Medicalization and conceptual disruption

Medicalization refers to processes of “defining more and more aspects of life in relation to aims defined within the medical domain” [24, p. 28]. Multiple authors describe that increasing possibilities to detect disease risk factors in early phases can contribute to medicalizing these early risk states (e.g. [14, 24, 32]). [see Table 2, column Medicalization & conceptual disruption]. They point to an ongoing trend that early detection can reinforce widening classifications of disease due to a focus on health and risk factors. Increased attention to early risk factors can reconceptualize what is regarded as “health” and “disease” and turn (what we consider now as) healthy people into patients in need of medical attention [21, 28, 53, 64].^3^ Improved understanding of the causes of disease can contribute to a blurring of the distinction between risk factors and disease indicators, thereby undermining the distinction between primary and secondary prevention and driving medicalization [32]. This may well change established views of what is normal or acceptable (e.g. in food habits) and thus interfere with sociocultural practices and values that are central in common conceptions of the good life, e.g., in relation to nutrition, lifestyle, or dignified aging [24, 47, 62].

The aim of detecting disease risk factors in an early phase to intervene and prevent disease development suggests that early disease is slumbering in everyone and must be “intercepted before it can strike” [37, p. 13]. This might cause feelings of agitation and insecurity as “*feeling* healthy no longer means *being* healthy” (23, p. 37, emphasis in the original). This reconceptualization of the meaning of being healthy reinforces assumptions that continuous (self-)monitoring is required, feeding into what David Armstrong [71] has dubbed “surveillance medicine”: the surveillance of healthy populations that dissolves the distinctions between health and illness and widens the space in which medicine operates [28, 37].

The increasing focus on early detection of risk factors and widening what is “actionable,” e.g., through innovations in medical practices and increasingly sensitive technologies for detecting biological “abnormalities,” is discussed as a driver for overdiagnosis and overtreatment [28, 36]. Overdiagnosis applies in “situations where an actual disease or risk factor is diagnosed in people who are mostly well, and where this condition will not actually come to influence future health, either because it disappears spontaneously without medical attention or remains asymptomatic until death from other causes” [28, p. 111]. Overdiagnosis and related overtreatment can be considered harmful if the disease would not have occurred anyway, if scarce healthcare resources are used, or if the medical interventions have serious physical and psychological sideeffects [28, 35, 39].

Finally, the increasing technological opportunities to obtain early risk information are thought to further contribute to medicalization and the above-discussed implications. In addition, worries were presented that commercialization of preventive health tests may lead to individuals screening themselves without proper understanding of the consequences [36], unrealistic expectations about tests’ explanatory power due to advertisements that make exaggerated promises about health protection [42, 72], increasing healthcare disparities due to inequalities in access, and decreasing health solidarity due to a further increase in individual responsibilities for health [72].

## Discussion

In this review we surveyed 55 articles and identified eight themes for the ethics of early detection of disease risk factors. The themes autonomy, beneficence & non-maleficence, and justice correspond to the bioethical principles from Beauchamps and Childress [58] and concern familiar ethical tensions between doing what is best for the individual patient and supporting their freedom to make their own choices (i.e. between beneficence & non-maleficence and autonomy), as well as between individual versus societal harms and benefits (i.e. between beneficence & non-maleficence and justice). We chose to discuss them separately for the sake of conceptual clarity, but frequently the concerns that were discussed in relation to these principles touched upon more than one principle at a time. Furthermore, no claims were made about the importance of the separate themes for distinct diseases or situations as we aimed to identify the broad range of ethical concerns that arise with (various forms of) early disease risk factor detection.

The other five themes, viz. reliability and uncertainty in early detection, privacy, downstream burdens on others, responsibility, and medicalization and conceptual disruption, also have links to the bioethical principles (e.g., the principle of non-maleficence could be used to support an argument against medicalization insofar as medicalization leads to harms [73]) but cover a wider spectrum of concerns that arise with early detection of disease risk factors [73]. These include harms that are not directly concerned with one’s physical state of being (e.g., privacy violations), potential shifts in societal practices (e.g., changes in the relative weight of health solidarity in public health debates), and, linking to recent work on social and conceptual disruption (cf. 74–76), changes in how concepts related to health and disease are understood.

### Gaps in the literature

Having an overview of these themes also lays bare certain concerns that are not as well represented in the literature, and which may be considered ‘literature gaps’. First, we observed a relative scarcity of research on ethical considerations associated with environmental preventive health interventions. This was surprising given that our search strategy explicitly included keywords related to exposures and the environment (see Supplemental materials). Focusing on risk factors and interventions within the environment is discussed as a necessary counterpart to detecting individual risk factors and the introduction of individual intervention strategies. It is also proposed by some as a solution to the current dominant focus on individual risk strategies and responsibilities. Nonetheless, there is little in-depth discussion of the ethical considerations for preventive interventions in the physical (shared) environment. Of particular interest would be analyses of potential stigmatization of specific living environments or neighborhoods [9], discussions on how to justify what parts of the environment are relevant to human health and in need of research or policy attention [76], and examinations on how to balance the promotion of human health with other values such as species preservation, ecological systems and landscape aesthetics [77, 78]. These subjects are particularly relevant if there is to be a shift towards utilizing or changing (aspects of) the environment with the aim of preventing and managing disease. We plea for more attention in bioethics for the ethics of environmental health interventions.

Second, we found that many authors have concerns about how overestimating individuals’ capacities for processing complex health information can lead to harmful effects such as misunderstanding risk information, feelings of stress and anxiety, and feeling overwhelmed. Such effects run contrary to the aim of empowering people to use (personalized) risk information to improve their health behaviors and lifestyle to protect their future health. However, relatively few authors make suggestions for how to improve the situation. This is worrisome because significant discrepancies between the capacities for making informed, reasoned, and voluntary health decisions that are presupposed by healthcare or governmental agencies and the capacities people have in actuality can leave people structurally falling short of expectations. When such “autonomy gaps” [79, 80] are not recognized and addressed, social and health inequalities can arise or widen as some individuals and groups are structurally disadvantaged regarding the development of the relevant capacities [80].

When authors do make suggestions, their solutions mostly focus on enhancing individuals’ capacities, for example through educational campaigns [41, 49]. Autonomy gaps, however, can also be reduced by adjusting institutional expectations. Autonomy gaps are unequally distributed along socioeconomic gradients and strategies to enhance individual capacities are not always effective or do not reach the most vulnerable groups [80]. When expectations are unnecessarily high and strategies for supporting individual capacities are ineffective, or worse, detrimental, lowering institutional expectations and creating more feasible health policies offers another approach to tackling autonomy gaps. We recommend prioritizing the identification of the sources of autonomy gaps in relation to early (public) health screening and the development of a wider set of possible mitigation strategies. Understanding how such gaps arise and may be resolved is also key for decreasing existing inequalities between those who are high and low in health literacy competencies.

Third, strategies for detecting individual risk factors and individual-level preventive interventions are also feared to lead to a systematic shift towards attributing more responsibility for health and disease to individuals. These concerns relate to discussions in the field of sociology on the *responsibilization* of (public) health [81–84]. Responsibilization refers to shifts in responsibilities from authorities to communities or individuals who are then presumed to take an active role in resolving their own problems [85–87]. However, many structural social and environmental factors that underly health risks at the population level are largely outside individuals’ control. Examples are air pollution or other harmful toxins in the environment, advertisements persuading the consumption of harmful products such as tobacco or fast food, or societal and peer pressure. Authors warn that responsibilization may direct attention away from more structural solutions and responsibilities by governments, industries, and employers.

Here too, however, we observed that relatively few authors make suggestions for how to improve the situation. A notable exception is the article by Stol et al., [72] that proposes a set of guiding principles and conditions for the ethical evaluations of (commercial) preventive health checks to counteract over-responsibilization. The paper also offers proposals for implementation by governments, healthcare institutions, and commercial companies. An overlooked complication with many environmental preventive strategies is that it is often difficult, if not impossible, to determine causality between environmental factors and disease outcomes and to disentangle the different causal roles of individual lifestyle factors, genetics, and environmental factors [6]. In addition, for healthcare and policy strategies it may be easier to tackle environmental risk factors by treating their effects in individuals, rather than tackling the environmental factors themselves, even though the latter can be more effective [88].

Pointing to issues of responsibilization is not meant to signal that responsibility for health needs to be attributed exclusively to either individuals or to the government. There are many different grounds for why individuals and institutions have obligations to promote and protect health and these are not necessarily in tension [89]. The reviewed body of literature shows serious concerns that early detection of disease risk factors will lead to assigning too much individual responsibility for health and disease and risks to shift attention and responsibility away from e.g. governments, industries, and employers. A first step to countervail this possible effect is to acknowledge that responsibility for health is not a zero-sum game: more responsibility for one actor does not imply less for another. Moreover, a key element for the development of health policies will be to reflect on (and ensure) a fair allocation of institutional responsibility for health [89].

Fourth, we found only one author who differentiated between screening programs in high-income and middle/low-income countries. Salamanca-Buentello et al., [34] discuss ethical considerations of screening for mental health in children and adolescents in the developing world. They point out that tools and approaches for early screening based on Western understandings of a disease may overlook local indicators of disease or mislabel and pathologize normal or culturally accepted behavioral variations. Therefore, screening instruments should be adaptable to non-Western contexts and validated in these contexts based on an appropriate scientific evidence base. Furthermore, the broader literature on ethics of prevention indicates that there may be distinct concerns about providing adequate healthcare support and access to treatment after detecting a highrisk [90], increasing global health disparities when prevention programs in developing countries cannot catch up with technological advances in developed countries [91], or differences in relevant moral values and their weighting [92]. Therefore, we recommend developing inclusive research strategies to obtain more non-Western perspectives on the ethics of detection of early risk factors. Doing so should help identify relevant commonalities and discrepancies that may feed into effective, context-specific policy decisions.

Fifth, we observed that in many articles, the ethical considerations that were discussed were not embedded in one or more normative frameworks in which the considerations could be evaluated. Though there are notable exceptions (e.g. [28]), we were often left to wonder during the review process how the wide variety of considerations at different levels should be balanced effectively and fairly. And while we readily acknowledge that it would be unrealistic to expect to find consensus in the literature about any specific normative position – Specker & Schermer [60] are even skeptical of the possibility of having a single evaluative framework – it would certainly help the field advance if authors were to embed their considerations explicitly and more systematically in a normative framework. Doing so would help draw out different possible ways of evaluating certain issues. For example, questions about the permissibility of particular public health interventions may be answered differently depending on whether one holds that interventions should enhance every person’s capacity to make healthy choices, or whether they should aim to maximize the overall health of the population.

Sixth, and finally, we observed a gap in connecting the considerations around the expected cost benefits of early detection (see the section Justice) to the prospective economic costs related to medicalization. It is tempting to think that “an ounce of prevention is worth a pound of cure” but this is not obvious, also not in early detection and screening. The increasing possibilities and implementations of detecting early disease risk factors may contribute to expanding the scope of (preventive) medicine and thus result in a further medicalization of society. Healthy people will request or require more frequent health checkups, mental support, and various kinds of guidance in their health trajectories, and this may place serious demands on already scarce healthcare resources [73, 93]. The economic costs associated with those demands may be significant, especially as life expectancy – often while living with chronic diseases – is increasing. As the articles we surveyed did not explicitly consider these costs, we recommend running extensive prospective studies into the costs of preventive screening programs, as well as the preventive medicine such programs entail, including the downstream costs of medicalization. Examining in more depth the connection between shifts towards preventive health strategies and (potentially unrealistic) reallocations of funds and healthcare resources will be important for deciding the ways in which such transitions should be shaped.

### Limitations

Our search strategy was limited exclusively to academic records. As such, we may have missed relevant ethical considerations described in grey literature such as blog posts, newsletters, or whitepapers. However, given the breadth of ethical considerations we discerned in the surveyed literature, we believe the risk of having overlooked pivotal concerns for this reason is minimal. A potentially more impactful limitation is the fact that our search strategy was limited to only two languages, English and Dutch. As a result, we may have failed to include valuable non-Western perspectives on the ethics of early risk detection. Future work could address this limitation by expanding the scope of the search in collaboration with academic partners from other countries. Another potential limitation is that the focus of our search strategy is explicitly on ethics literature. It is possible that we may have missed policy documents or academic publications from neighboring fields in which ethical considerations around early screening practices were discussed in other, less explicitly ethical terms. While this narrower focus of the search strategy suited our research aim, and broadening the focus would likely not have affected the overall landscape of ethical considerations we found, it may potentially explain the relative scarcity of policy recommendations (see the section on Gaps in the literature). To test this hypothesis, future reviews in this area could include a secondary search strategy for other types of publications concerned with, for example, the legal dimensions of screening programs and early risk factor detection.

## Conclusions

Early detection of disease risk factors relates to many different but frequently intricately related ethical considerations. The motivation for performing this scoping review was the fragmented state of the ethics literature in this domain. By systematically surveying the literature, grouping the ethical considerations into eight themes, and identifying gaps in the surveyed literature, we have provided a fuller picture of the relevant kinds of considerations and their saliency in academic records.

The breadth of the considerations we identified speaks not only to the complicated nature of risk and risk information, but also to the wide-ranging implications that novel technologies for measuring, modelling, and analyzing increasingly large clusters of environmental factors and linking these to disease outcomes may have. Based on the present review, it may be surmised that scientific progress in understanding the long-term, interrelated effects of exposures over time (viz. of the human exposome) will have significant downstream effects that will raise challenging questions about how to (re)structure healthcare in ways that are individually and societally beneficial and economically viable. Two concluding remarks about this are in order. First, if responses to these challenges are to be properly informed, the present body of ethics literature should be expanded, not only with improved normative content, but also with more in-depth, detailed discussions about the expected upsides of early risk factor detection. Frequently, in the literature we surveyed, the upsides are passed over quickly in order to express ethical concern(s), but both perspectives are needed to strike a good balance. Second, given the rapid advancements in the field, especially with regards to methods of detection and methods of analysis [94], ethical analysis of new possibilities for early detection of risk factors is urgent. Timely reflection on ethical aspects may contribute to responsible and fair health policies.

### Electronic supplementary material

Below is the link to the electronic supplementary material.

Supplementary Material 1

## Data Availability

The datasets generated and analysed during the current study are available from the corresponding author on reasonable request. The search strings used to generate the datasets can be found in the supplementary information file.

## Footnotes

1 This is also known as the Prevention Paradox: “a measure that brings large benefits to the community offers little to each participating individual.” (Rose, 1981, p. 1850).

2 Throughout the manuscript, the term “authors” always refers to the authors of included articles discussed in that paragraph.

3 These “healthy people turned into patients” are also discussed as “patients in waiting” [12, 53], “healthy ill” [50] and “risk individuals” [28].
