# Supplementary material for: Ethics of early detection of disease risk factors: A scoping review
Source: BMC Med Ethics. 2024 Mar 5;25:25. doi: 10.1186/s12910-024-01012-4 (PMC10913641; doi:10.1186/s12910-024-01012-4)
Supplement: Supplementary file 1 — Supplementary Material 1 [file 12910_2024_1012_MOESM1_ESM.docx]

**Supplemental material**

File Name: Additional File 1

File format: Word document (.docx)

Title of data: Search Strategy for the Academic Literature

Description of data: Searches performed April, 5th, 2022 in the Scopus and Embase databases. Search performed December, 22th, 2023 in Philosopher’s Index.

Scopus

TITLE-ABS ( ethic* ) AND TITLE-ABS-KEY ( ( "Early detection" OR "Preclinical detection" OR "Predict*" OR "Prodrom*" OR precurs* OR prevent* OR primal OR primordial ) AND ( ( "Risk factor" OR "protective factor" OR determinant OR exposure OR biomarker OR indicator ) OR ( exposom* OR multi-expos* OR ( multi PRE/2 expos* ) co-expos* OR *omic OR epigen* OR environ* OR "lifecourse epidemiology" OR "Environmental epidemiology" ) ) ) AND NOT ( TITLE-ABS-KEY ( clinic* OR trial OR randomi* OR protocol OR animal OR rat OR mice OR "ethic* commit*" OR "ethic* approv*" ) ) AND ( LIMIT-TO ( LANGUAGE , "English" ) OR LIMIT-TO ( LANGUAGE , "Dutch" ) )

Embase

('ethics'/exp/mj OR ethic*:ti,ab) AND ('prediction'/exp OR 'prediction':ti,ab OR 'preclinical study'/exp OR 'preclinical study':ti,ab OR 'prodrome'/exp OR 'precursor'/exp OR 'early detection':ti,ab OR 'preclinical detection':ti,ab OR 'predict*':ti,ab OR 'prodrom*':ti,ab OR precurs*:ti,ab OR prevent*:ti,ab OR primal:ti,ab OR primordial:ti,ab) AND ('risk factor'/exp OR 'protective agent'/exp OR 'determinant'/exp OR 'environmental exposure'/exp OR 'biological marker'/exp OR 'risk factor':ti,ab OR 'protective factor':ti,ab OR determinant:ti,ab OR exposure:ti,ab OR biomarker:ti,ab OR indicator:ti,ab OR 'exposome'/exp OR 'exposomics'/exp OR 'exposome':ti,ab OR exposomic*:ti,ab OR 'multi expos*':ti,ab OR 'lifecourse epidemiology':ti,ab OR 'environmental epidemiology':ti,ab OR 'co expos*':ti,ab OR 'omics'/exp OR 'omics':ti,ab OR 'epigenetics'/exp OR 'epigenome'/exp OR epigen*:ti,ab OR 'environmental monitoring'/exp OR 'environmental surveillance'/exp) NOT (clinic*:ti,ab OR trial:ti,ab OR randomi*:ti,ab OR protocol:ti,ab OR animal:ti,ab OR rat:ti,ab OR mice:ti,ab OR 'ethic* commit*':ti,ab OR 'ethic* approv*':ti,ab OR 'ethic* and dissem*':ti,ab) AND ([dutch]/lim OR [english]/lim) AND [humans]/lim

Philosopher’s Index

((ethic* and ("Early detection" or "Preclinical detection" or "Predict*" or "Prodrom*" or precurs* or prevent* or primal or primordial) and ("Risk factor" or "protective factor" or determinant or exposure or biomarker or indicator or (exposom* or "multi-expos*" or omic or epigen* or environ* or "lifecourse epidemiology" or "Environmental epidemiology"))) not (clinic* or trial or randomi* or protocol or animal or rat or mice or "ethic* commit*" or "ethic* approv*")).ab,ti. 
